# A oneM2M-Based Query Engine for Internet of Things (IoT) Data Streams

**DOI:** 10.3390/s18103253

**Published:** 2018-09-27

**Authors:** Putu Wiramaswara Widya, Yoga Yustiawan, Joonho Kwon

**Affiliations:** 1 Department of Big Data, Pusan National University, Busan 46241, Korea; wira@pusan.ac.kr (P.W.W.); yoga017@pusan.ac.kr (Y.Y.); 2 School of Computer Science and Engineering, Pusan National University, Busan 46241, Korea

**Keywords:** IoT data streams, IoT data retrieval, query engine, oneM2M, hybrid infrastructure-edge processing, edge analytics

## Abstract

The new standard oneM2M (one machine-to-machine) aims to standardize the architecture and protocols of Internet of Things (IoT) middleware for better interoperability. Although the standard seems promising, it lacks several features for efficiently searching and retrieving IoT data which satisfy users’ intentions. In this paper, we design and develop a oneM2M-based query engine, called OMQ, that provides a real-time processing over IoT data streams. For this purpose, we define a query language which enables users to retrieve IoT data from data sources using JavaScript Object Notation (JSON). We also propose efficient query processing algorithms which utilizes the oneM2M architecture consisting of two nodes: (1) the IoT node and (2) the infrastructure node. IoT nodes of OMQ are mainly sensor devices execute user queries the aggregate, transform and filter operators, whereas the infrastructure node handles the join operator of user queries. Since the query processing algorithms are implemented as the hybrid infrastructure-edge processing, user queries can be executed efficiently in each IoT node rather than only in the infrastructure node. Thus, our OMQ system reduces the query processing time and the network bandwidth. We conducted a comprehensive evaluation of OMQ using a real and a synthetic data set. Experimental results demonstrate the feasibility and efficiency of OMQ system for executing queries and transferring data from each IoT node.

## 1. Introduction

In recent years, there has been a rapid deployment of a massive number of Internet of Things (IoT) devices  [1]. Currently, there are very large number of interconnected devices, and their numbers are still increasing and expected to reach 25 billion devices by 2020 [2]. For example, smart city applications require to install many IoT devices for monitoring city infrastructure such as roads [3], buildings [4], and streams [5].

One of the major challenges in IoT is the retrieval and processing of a large amount of heterogeneous streaming data generated from a large number of IoT devices [6,7]. Unlike traditional database management systems (DBMS) where data are structured in schema and their query languages are standardized, each IoT device has a different data retrieval method, data format, and application programming interface (API). This means that collecting data from several IoT devices can be complicated, especially for application developers who want to make use of IoT in their application.

Most IoT implementations exploit the functionalities of IoT middleware to effectively manage numerous IoT devices. An IoT middleware [8,9] provides an abstraction for the application developers to interface IoT devices without knowing any details or protocols behind them. However, due to the lack of collaboration among IoT industries, most existing IoT middleware does not have a common specification for the protocol and services for supporting a broader interoperability [10].

To overcome this fragmentation issue, several leading standards development organizations worked together to suggest a new standard called oneM2M is proposed for M2M (machine-to-machine) and IoT. The main objective of oneM2M is to minimize fragmentation at the M2M/IoT service layer fragmentation [11]. Thus, several pieces of oneM2M-based middleware such as Eclipse OM2M [12], Mobius [13] and Secure OM2M Service Platform [14] are developed and released as open source.

Since existing oneM2M-based middleware focuses on the fragmentation issue or the security issue, they do not provide essential features for searching and retrieving IoT data streams. More specifically, they lack content-based data processing such as filtering and other basic operations for retrieval. Also they do not support any metadata-based search mechanisms. We believe that a oneM2M-based query engine is necessary because it provides a way for IoT application developers to define what data they want to use, and how the data should be processed in a single query language. Thus, with the increased the abstraction of oneM2M-based query engine, developers could focus on what they have to do with the data.

In this paper, we propose a oneM2M-based query engine, called (OMQ). The main goal of OMQ is to provide crucial query processing functionalities for IoT applications on top of oneM2M middleware. The main contributions of this paper can be summarized as follows:

- We propose a oneM2M-based query engine for efficiently searching and retrieving IoT data streams.
- We define a JSON-based query language which enables users to specify data source search metadata properties and execution operators.
- The architecture of OMQ facilitates on-demand multiple ad-hoc queries and the efficient execution of hybrid infrastructure-edge query processing algorithms.

The remainder of this paper is organized as follows. Section 2 presents related research work. Section 3 provides some brief preliminaries regarding oneM2M standard and how it works. Section 4 explains OMQ architecture and Section 5 describes the OMQ implementation and its performance evaluation. Finally, we conclude the paper in Section 6.

## 2. Related Works

### 2.1. oneM2M Middleware

The oneM2M standard defines some common service functions (CSFs) that an IoT/M2M service or middleware has to comply. The goal of the standard is to provide a common functionality to simplify the application development and remove the need to develop common components [11]. The two basic CSFs include the ability to register, discover and control devices, and manage the data coming from a device to the user through a subscription model. There are several pieces of oneM2M-based middleware. One open-source middleware is called Mobius [13], a Node.js oneM2M middleware that provides wireless-sensor integration. Another open-source-based middleware is called OM2M [12], which was originally based on ETSI standard but is currently modified to comply with the oneM2M standard. In Korea, a proprietary oneM2M-compliant middleware such as HANDYPIA [15], has a unique semantic-enabled IoT middleware feature, whereas SKT ThingPlug (https://sandbox.sktiot.com/IoTPortal/main/changeLocale?locale=en) and Olleh IoTMaker (http://iotmakers.olleh.com/openp/index.html), provide a cloud infrastructure that users can utilize to host their middleware.

**Figure 1:**
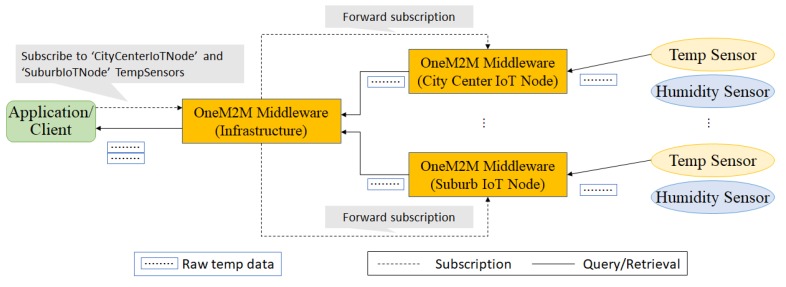
Current oneM2M-based middleware approach for data retrieval [13].

Figure 1 illustrates the current approach of existing IoT middleware [13]. When an IoT application requests to obtain a specific stream data, it should create a subscription to a specific data source URI via the middleware. However, an IoT application of the current approach cannot specify any filtering and processing operations in the subscription; thus, it has to retrieve all raw data and to apply further processing by itself. Consider Figure 1 again. Assume that the client application wants to subscribe all the temperature raw data from two different IoT nodes located at a city center and a suburban area. In this case, an IoT application should include the exact URI location of the data source they wish to subscribe since the current oneM2M middleware does not provide any metadata-based search mechanisms.

**Figure 2:**
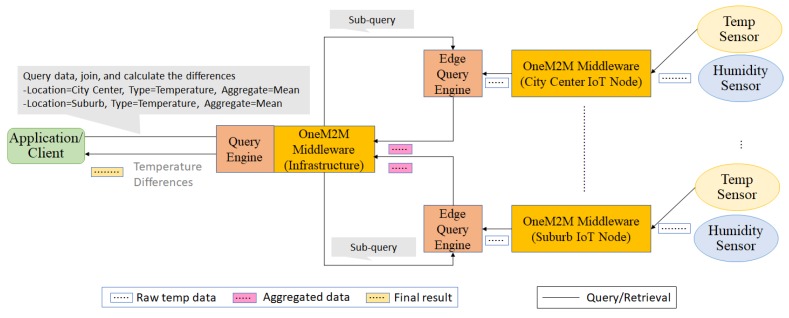
Our proposed query engine (QE) for better oneM2M data retrieval and processing.

OMQ addresses the two features lacking in oneM2M-based middleware as shown in Figure 2. First, OMQ adds a query-based data processing functionality that addresses the lack of content-based data processing in oneM2M [16]. A client or an application can utilize OMQ to process the raw data using a processing operator such as aggregation, which can reduce the bandwidth usage significantly. Second, OMQ provides a searching feature that searches the data source based on the given metadata property (such as sensor type or sensor location), then a client does not have to know the exact physical location to obtain specific data. The current implementation of oneM2M middleware (including Mobius) provides only a simple discovery feature that list all data sources without any filtering mechanism. Another benefit of implementing a query engine (QE) on top of oneM2M is that it can be implemented and integrated easily on various oneM2M-compliant middleware.

### 2.2. IoT Data Processing

Due to the multi-layered and heterogenous nature of IoT implementation, the IoT data processing researches can be divided into three categories: infrastructure-based processing, edge-based processing, and hybrid processing. In infrastructure-based processing, IoT data are processed by a cluster of high-capacity, high-performance servers located in either the private infrastructure or the public cloud. There are several so-called “Big Data” technologies for this type of data processing such as Apache Hadoop, which provides Map-Reduce [17]-based batch-processing; Apache Spark [18], which provides in-memory batch and stream processing; and Apache Storm [19], which provides a graph-driven stream-processor. However, processing data exclusively in the infrastructure involves sending all raw data from the IoT nodes to the infrastructure, which can increase network bandwidth and latency. In edge-based processing (also known as “edge analytics”, “fog computing”, or “edge computing”), IoT data is processed on edge devices such as sensor node or gateway node. Because data are processed on the same network where it produced, it can process data without any network latency. This edge-based processing is important in real-time, mission-critical applications such as self-driving cars [20]. WSN query processing systems [21,22,23] are some examples of edge-based processing where processing is performed right in the sensor node. In other types of edge-based processing, the processing is performed in the gateway-level [24,25,26,27]. Even though the current generation of edge devices (such as Raspberry Pi, PandaBoard) possess a high computing throughput, it still cannot challenge the performance of the infrastructure server. Therefore, in general edge-based processing is usually done mainly for data pre-processing purposes to reduce the bandwidth and storage usage for sending and storing IoT data.

To compensate the lack of processing power in edge devices, some researchers have suggested a hybrid-approach, where some processing can be done in the edge devices and the remaining (usually more complex) processing is done in the infrastructure. Hu et al. [28] proposed combining edge-processing system Global Sensor Network (GSN) [25] with Storm. Govindarajan et al. [29,30] suggested an automatic way to split a given graph of queries into two sets: a set of connected queries that will be processed in the edge devices and another set of queries that will be processed in the cloud. ECHO [31] offers an application lifecycle manager that receives JSON-based dataflow information and provisions different types of data processing platforms in a variety of edge devices and cloud accordingly.

As depicted in Figure 2, our OMQ accepts ad-hoc queries written as JSON-based text files through a standardized query language. The query language consists of (1) data sources definition that define the data source metadata properties, and (2) query processing operators such as filtering, aggregation, transformation, and join. OMQ also supports simultaneous processing of multiple stream-based queries, where an on-demand query can be started and stopped at any time. Finally, OMQ supports hybrid edge-infrastructure processing by splitting a given query operator automatically into two parts: one part that will be processed by the edge node, and another part that will be processed by the cloud or fog infrastructure.

## 3. Introduction to oneM2M Standard

The oneM2M standards are specified in several published specification documents (http://www.onem2m.org/technical/published-documents), which include functional architecture [32], service layer protocol [33] with different bindings (HTTP or MQTT), and some auxiliary documents regarding internetworking, security, and applications. One main aspect of oneM2M standards is the separation between the application entity and common service entity. An application entity (AE) is a software or program that is responsible for executing the application logic of an IoT or M2M system. Some examples of AE include sensor reading, actuator control, device monitoring, or power metering. A common service entity (CSE) provides some common services collectively referred to as CSFs, which include data management, device management, subscription management, and location services. These services can be utilized by AEs or other CSEs.

**Figure 3:**
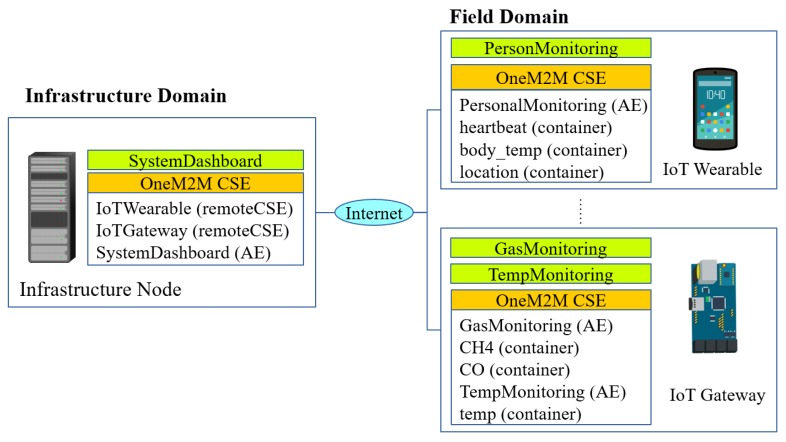
An example of oneM2M-based IoT network.

Figure 3 illustrates an example of oneM2M-based IoT network. Two main domains of oneM2M-based IoT network are: (1) an infrastructure domain and (2) a field domain. The main IoT service provider usually resides inside the cloud infrastructure. Thus, the infrastructure domain consists of one or several infrastructure nodes (IN). An infrastructure node contains one CSE and several AEs. Examples of application entities in the infrastructure domain include a command center application, a monitoring application, and others. The field domain is a site or a field where IoT nodes resides. IoT nodes include several constrained devices such as sensors, actuators or IoT gateways.

Another important aspect of the oneM2M standard is that each CSE has a standardized representation called resource tree. This tree represents the resources or entities that a CSE handles. An AE resource type defines name, point of access, and other attributes corresponding to an AE. A container defines a data source container that can be used by an AE to get/put data from/to another AE through a publisher or subscriber model. A remote CSE defines an associated remote CSE that can be accessed remotely. To manipulate a resource tree, oneM2M provides a REST-based API that can be used to create, read, update, or delete a resource. Consider again Figure 3. To refer to the CH4 container of the IoT Gateway node, we can access it through the infrastructure node via URL /IN-CSE/IoTGateway/GasMonitoring/CH4. This API can be accessed through several protocol bindings: HTTP, COAP, WebSocket, or MQTT.

## 4. Proposed System Architecture

**Figure 4:**
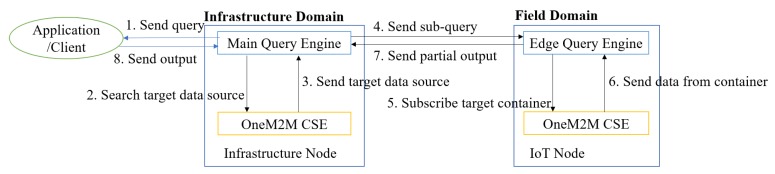
The proposed oneM2M query engine.

The proposed OMQ system is depicted in Figure 4. The QE is installed inside an infrastructure node located in the infrastructure domain. To support hybrid edge-infrastructure processing, an edge QE can be installed on some of the IoT nodes. The QE receives a query *q* from a client (such as applications, users or web dashboard) to be processed. Before processing a query, the QE will search for the IoT node and data source that matches the given data source property defined inside the query. This process involves a communication between the QE and oneM2M CSE. After the target data sources are resolved, the main QE then splits the query into sub-queries and sends those sub-queries into all related IoT nodes that host the target data sources.

### 4.1. Query Language Definition

OMQ receives a query q={n,D,O}, which consists of *n* data sources, data sources definition $D=\{d^{\left(1\right)},d^{\left(2\right)},\cdots ,d^{\left(n\right)}\}$, and a set of query operators O. A data source definition $d^{\left(x\right)}$ is a set of data source properties a user wishes to query in the form of a key-value pair. A query consists of several connecting operators $O=\{O_{prejoin},o_{join},O_{postjoin}\}$, which are divided into three parts:

- **Pre-join operators**$O_{prejoin}=\left\{O_{prejoin}^{\left(1\right)},O_{prejoin}^{\left(2\right)},\cdots ,O_{prejoin}^{\left(n\right)}\right\}$, where $O_{prejoin}^{\left(x\right)}=\left\{o^{\left(1\right)},o^{\left(2\right)},\cdots \right\}$ defines a set of chaining query processing operators that will be applied for each corresponding data source as pre-processing steps before joining the data into a single tuple.
- **A join operator**$o_{join}$ defines a join function that joins the inputs from several data sources into one tuple.
- **Post-join operators**$O_{postjoin}=\left\{o^{\left(1\right)},o^{\left(2\right)},\cdots \right\}$ defines a set of chaining operators applied to the data after being joined into a single tuple.

Table 1 lists all the operators supported by OMQ. Both pre-join and post-join operator chains can consist of one or multiple combinations of these operators, except the join operator, which can only be defined once in a single query.

**Figure 5:**
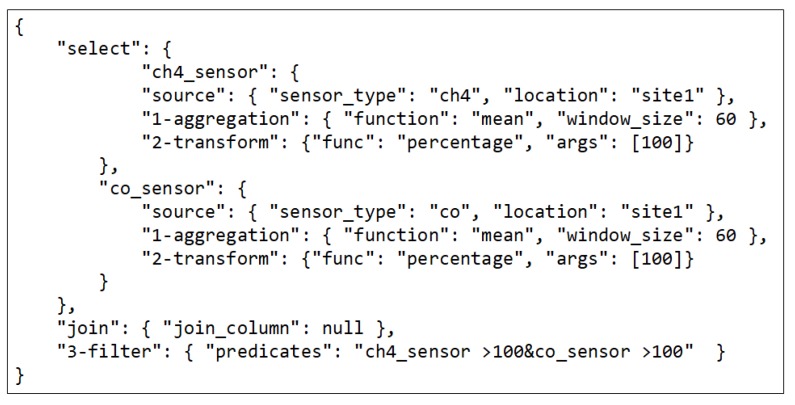
An example query.

**Figure 6:**
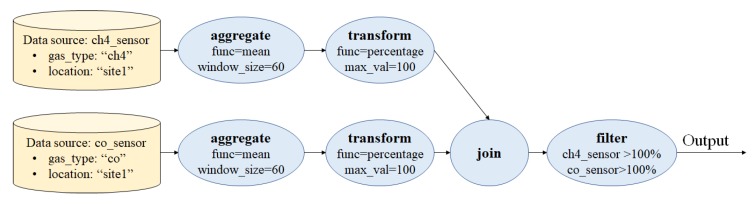
A query DAG of corresponding query shown on Figure 5.

Users can specify their intentions as queries using a JSON-based format as shown in Figure 5. Here, the example query consists of two data sources: $d^{\left(1\right)}=\left\{\left(\mathrm{sensor}\_\mathrm{type},\mathrm{ch}4\right),\left(\mathrm{location},\mathrm{site}1\right)\right\}$ and $d^{\left(2\right)}=\left\{\left(\mathrm{sensor}\_\mathrm{type},\mathrm{co}\right),\left(\mathrm{location},\mathrm{site}1\right)\right\}$ with pre-join operators $O_{prejoin}^{\left(i\right)}=\left\{o_{aggr}^{\mathrm{func}\;=\;\mathrm{mean},\mathrm{winsize}\;=\;60},o_{trans}^{\mathrm{func}\;=\;\mathrm{percentage},\mathrm{maxval}\;=\;100}\right\},i\in \left\{1,2\right\}$ for each data source respectively. The join operator is defined as $o_{join}^{\mathrm{type}\;=\;\mathrm{timestamp}}$, and the post-join operators are defined as $O_{postjoin}=\left\{o_{filter}^{\mathrm{ch}4\_\mathrm{sensor}\;\geq \;1,\mathrm{co}\_\mathrm{sensor}\;\geq \;1}\right\}$. Another way to represent this query is through a query directed acyclic diagram (DAG), which is shown in Figure 6. For the given example query, the QE will aggregate each sensor data input using the same aggregation function (mean) and same window size (60 s). Then, each aggregated data is transformed into a percentage. After being processed separately, each data source will then be joined into a single tuple based on a similar timestamp. After joining, both data are filtered, so that it only outputs when both the readings are 100%.

The query format is inspired from the GSN virtual sensor definition [25]. According to the GSN virtual sensor definition, a user can define several data sources consisting of key-value data source properties and a query statement, which is applied on each data source. Then, in the same file, a user can apply a query on the whole joined data from all defined data sources.

### 4.2. Query Engine Architecture

**Figure 7:**
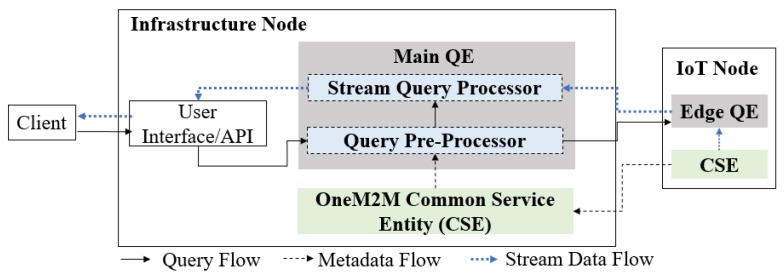
oneM2M QE architecture.

Figure 7 shows a more detailed schematic of the QE architecture inside OMQ. A QE consists of two modules: *query pre-processor*, which performs query planning tasks, and a *stream query processor*, which performs the stream processing according to a given query. Each QE is connected with oneM2M CSE for communicating between the data sources and the query processor. The main QE is also connected with the edge QE located inside several IoT nodes in the field domain. The edge QE uses mostly the same part as the main QE, with the query pre-processor part removed, as the pre-processing is done mainly in the infrastructure side.

#### 4.2.1. Query Pre-Processor

**Figure 8:**
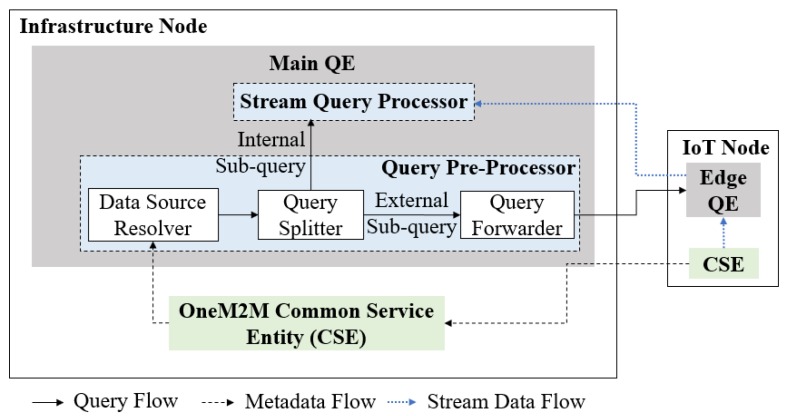
Query Pre-Processor module.

The query pre-processor consists of three main components: *data source resolver*, *query splitter* and *query forwarder* as depicted Figure 8. The input query firstly goes into the *data source resolver* module in the infrastructure node. This module maps each data source search property (denoted as $d^{\left(x\right)}$) of a query *q* into a corresponding oneM2M container URI. This mapping process can be achieved by matching *d* with the key-value metadata information of all the containers in a resource tree.

To achieve a fast mapping, the data source resolver maintains a table data structure called *metadata mapping M* for avoiding scanning all the resource trees. During a startup, the module builds *M* by discovering the related resources using Algorithm 1, which starts from the scanning container in the infrastructure node’s CSE. Then, if a remote CSE is registered inside the CSE, the module will also recursively scan those remote CSEs for available containers. the *data source resolver* module quickly transforms a given data source search property of a new incoming query *q* into several key-value pairs with a matching container URI by checking the constructed *M*. It also updates *M* periodically to reflect the current state of the system.

**Algorithm 1:** Building metadata mapping for data source resolver  **Input**: nodeIP: physical IP address of node, starting from IN  **Output**: *M*

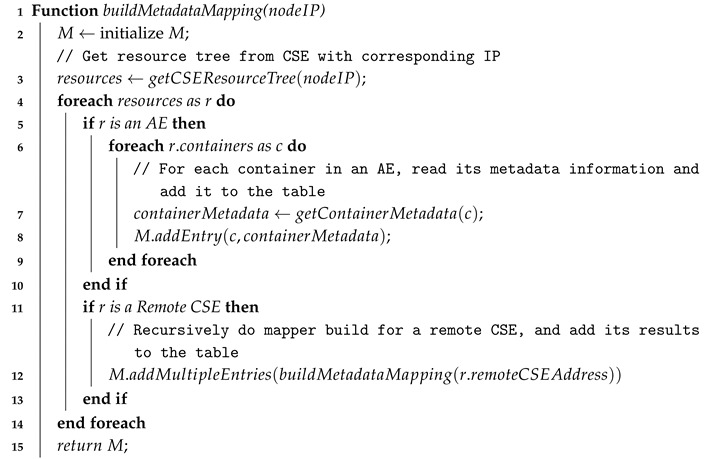

**Figure 9:**
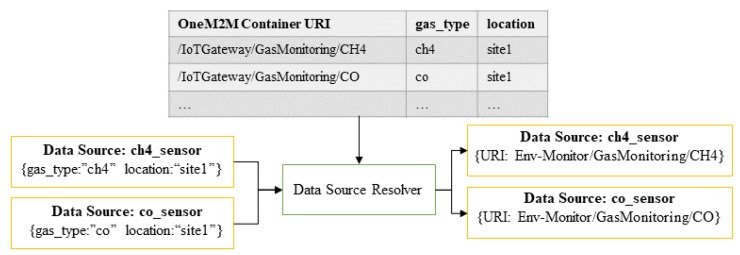
An illustration of data source resolver.

**Example** **1.** *Figure 9 explains how the data source resolver works. Two data sources with each search property are obtained from two different sensors such as ch4_sensor and co_sensor. Each data source is mapped quickly to the corresponding oneM2M container URI by checking metadata mapping table M. The data source resolver outputs each data source with the corresponding matching container URI information.*

After resolving the target container URIs of each data source definition, the query will then pass into the *query splitter* module. This module splits a query *q* into two sub-queries: (1) *internal sub-query* $sq_{int}$ which will be processed in the IoT node and (2) *external sub-query* $sq_{ext}$ which will be processed in the infrastructure node.

The pseudo code for query splitting is shown in Algorithm 2. For each data sources in a given query *q*, the query splitter will check whether the node that hosts the corresponding data source has its own edge QE installed. If it does, an instance of $sq_{ext}$ for the corresponding edge QE will be created with all the pre-join operators of the corresponding data source included as its operators. Otherwise, the data source and its pre-join operators will be included in $sq_{int}$ to be processed in the main QE. Note that join and post-join operators will always be included as part of $sq_{int}$.

**Algorithm 2:** Query Splitting Algorithm   **Input**: *q*: a query input   **Output**: $sq_{int}$ internal sub-query, $sq_{ext}$ external-subquery

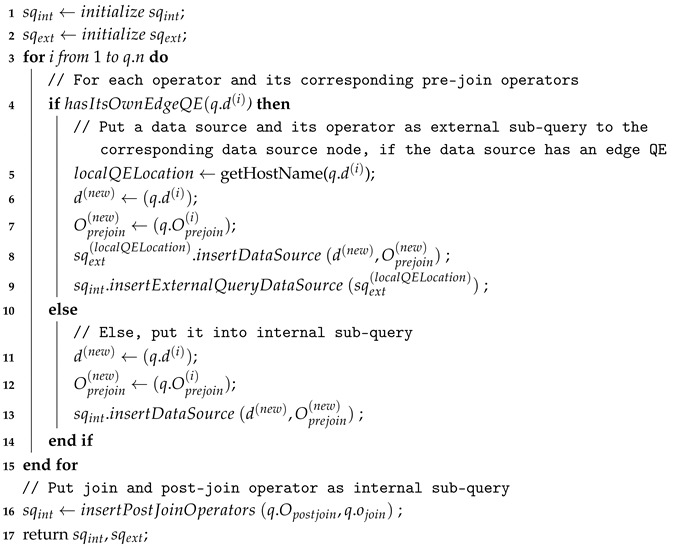

**Figure 10:**
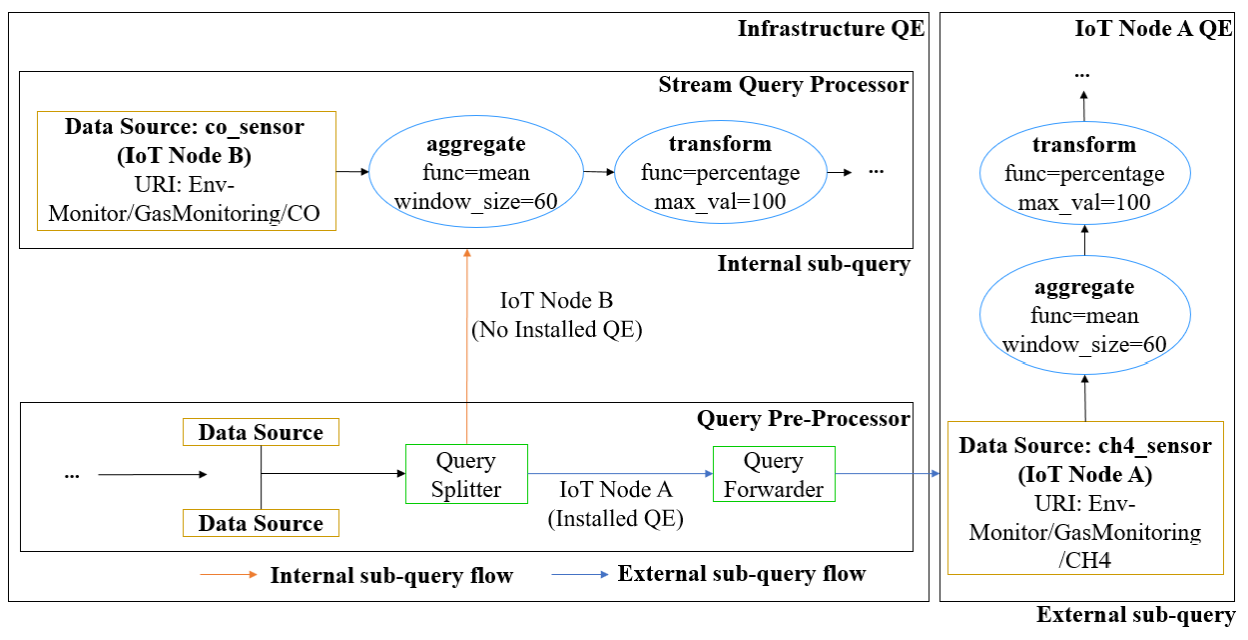
Query splitting demonstration.

**Example** **2.** *Figure 10 depicts the internal and external sub-query splitting results from the original query shown in Figure 6. The user query contains aggregation and transformation operations for the two data sources ch4_sensor and co_sensor. The aggregation operation is specified with a mean function and a sliding window of size 60 seconds, whereas the transform operation is specified with a percentage function and maximum value 100. These two data sources with the container URI information go to the query splitter.*

*Based on the sub-query for data source ch4_sensor, the query splitter checks the corresponding IoT node whether it has its own QE or not. Since the corresponding IoT node A for ch4_sensor evidently has its own QE, then the sub-query is forwarded by query forwarder to the IoT node A. This is a case for the external sub-query. For another sub-query for data source co_sensor, the corresponding IoT node B is checked by query splitter. It turns out that the IoT node B does not have its QE. Therefore, this sub-query is directly forwarded to the stream query processor module and handled by the QE of the infrastructure node. This is a case for the internal sub-query.*

The *query forwarder* module mainly delivers the external sub-query from *query splitter* to each corresponding IoT node. Since it only forwards the external sub-query, this module is used only when there exists any corresponding IoT node with its own QE.

#### 4.2.2. Stream Query Processor

**Figure 11:**
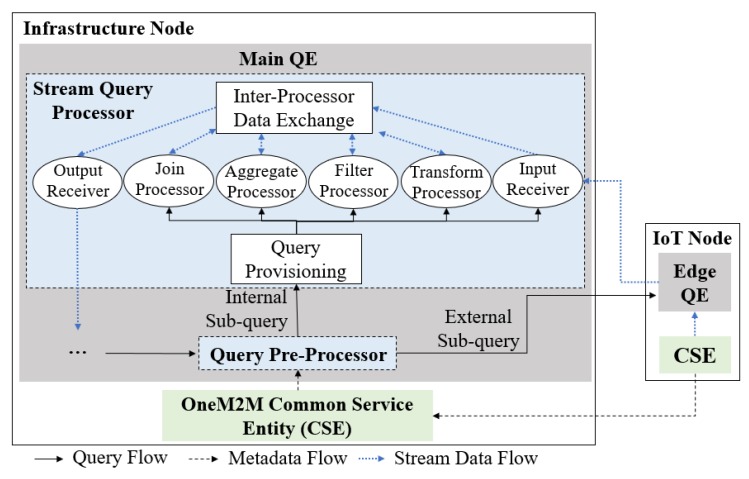
Stream Query Processor module.

The stream query processor module consists of several *processors* that are responsible for four basic query operators, two additional processors (input and output receiver), query provisioning, and inter-processor data exchange, as depicted in Figure 11.

The *input receiver* module receives the input data stream as either raw data from the CSE data source or as partially processed data from an edge QE, later moves to the first defined operator according to the current running query. Each processor handles the given input stream according to the given query operator. Then it places the processed stream data as its output and provide it to another stream which will be handled by the next operator. This data processing is done for each chaining operator. When the data is handled by the last operator of the chain and the results are sent to the *output receiver* module. The *output receiver* module reports the final results to the user through the *user interface*. This process will stop based on user demand, otherwise it continuously run.

Both main QE and edge QE have a similar stream query processor architecture; however, the latter does not include the join processor because the join processes are performed only in the main QE.

To control how a processor processes an input data, each processor maintains a data structure called *process mapping*. Each entry of the process mapping contains a tuple of (input stream ids, operator definition, and an output stream id). All the streams are identified by a unique string-based identifier. The aggregate, transform and filter processors accept only a single input stream, whereas the join operator can accept multiple input streams at once. The input receiver annotates the data input by adding a stream identification (named ch4_sensor and co_sensor).

**Figure 12:**
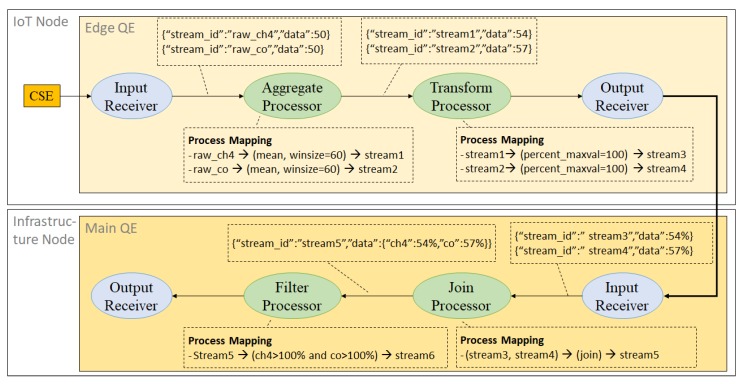
An illustration of data processing inside stream query processor.

**Example** **3.** *Figure 12 illustrates the data processing steps needed to process sub-queries from Figure 10. The edge QE receives two data inputs from the two sensors (CO and CH4) directly from the CSE located on the same node. The input receiver annotates the input data by adding a stream identification (named ch4_sensor and co_sensor).*

The *inter-processor data exchange* module aims to determine the operator’s processor that will be responsible for processing the given stream data. Algorithm 3 explains how the input data stream data are handled inside the operator’s processor. When one of operator’s processors takes the input data stream, this processor firstly checks its process mapping table by matching the corresponding data stream identifier (id) to determine how the stream data is going to be processed.

**Algorithm 3:** Data processing inside an operator’s processor  **Input**: inputStreamData: Input stream data  // This procedure is called whenever an operator’s processor get a new input data stream

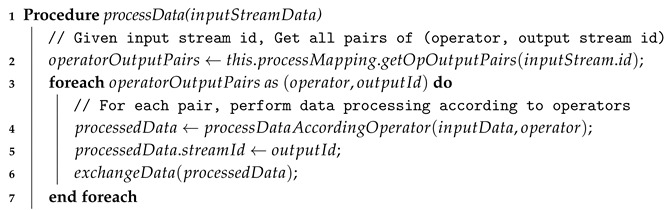


If the input data stream identifier exists in the process mapping table for this operator’s processor, the *inter-processor data exchange* module forwards this data stream to the defined operator’s processor, as described in Algorithm 4.

**Algorithm 4:** Inter-operator data exchange  **Input**: streamData: Input stream identifier
  // This procedure is called whenever the inter-operator data exchange receives processed data from an operator’s processor

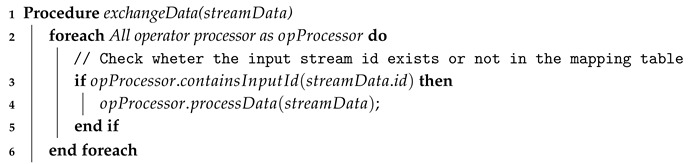


The operator processors for aggregate/join/filter/transform as shown in Figure 7 handle the input stream data to compute the results. They send the output results to the *inter-processor data exchange* module. The operator’s processor ends when the *inter-processor data exchange* module forwards the processed stream data into the *output receiver* module. The processed stream data are sent to the user clients or to the main QE for post-join operator processing.

The stream query processor supports simultaneous handling of multiple queries through the *query provisioning* module in Figure 7. This module receives a sub-query submission either as an internal sub-query or as an external sub-query. It aims to modify the processor mappings of several related operators when it takes a new sub-query sq as an input. This module also creates the process mapping for each operator’s processor based on the input sub-query. However, it only creates the process mapping for join operator’s processor when the input sub-query is an internal sub-query.

Algorithm 5 shows the pseudo code for the query provisioning. It scans through all pre-join operators and post-join operators within the input query. One of key action of Algorithm 5 is to create a mapping for an operator’s processor. Algorithm 6 explains how a new process mapping entry is added. Before adding a new entry into a process mapping, the query provisioning has to make sure if the operator processor has already processed the same combination of (input stream identifier, operator parameters) in order to maximize data and operator sharing. Otherwise, it creates a new entry in the process mapping. 

**Algorithm 5:** Query provisioning **Input**: sq: sub-query // This procedure is called whenever a new query need to be provisioned inside a QE

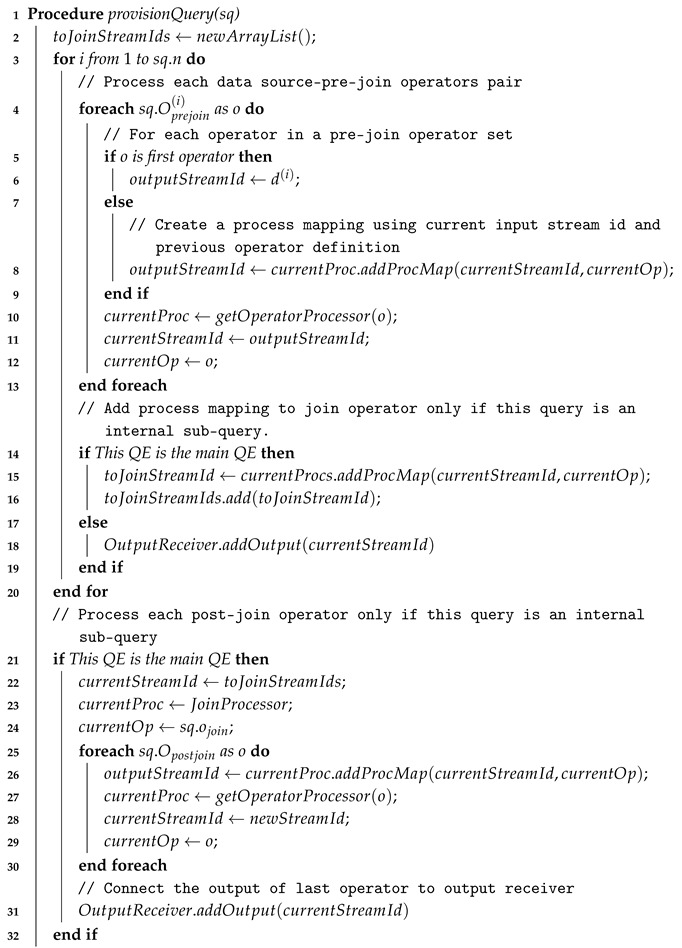


**Algorithm 6:** Add process mapping to an operator processor **Input**: inputStreamId: Input stream identifier
 **Input**: op: Operator parameters
 **Output**: outputStreamId: Output stream identifier
 // This function is called when the query provisioner try to add an entry into one of operator processor’s process mapping table

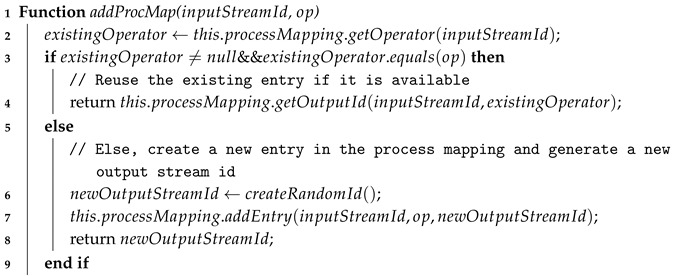


## 5. Evaluation

### 5.1. Software Implementation

We implemented all the QE components including the query pre-processor and stream query processor modules using Java Development Kit (JDK) version 1.8.0 (Oracle Corp., Redwood City, CA, USA, 2017). We utilize a multi-threading approach by implementing each operator processor as a single separate thread. For data communication among QEs, we utilize a message queue server using Rabbit MQ 3.6.10. We exploit Mobius YellowTurtle version 2.3.8 (Open Alliance for IoT Standard (OCEAN), Seongnam, Korea, 2017) and nCube Rosemary version 2.1.14 (Open Alliance for IoT Standard (OCEAN), Seongnam, Korea, 2016) (both are based on the same Mobius codebase) for oneM2M CSE implementation in the infrastructure node and IoT node respectively. The query engine (QE) communicates with CSE through REST-based HTTP endpoint. The implementation code of the OMQ system can be acquired in [34].

### 5.2. Experiments

We evaluated the OMQ using two different scenarios. In the first scenario, we performed a benchmark test on the stream query processor using a synthetic load. In the second scenario, we implemented the QE on top of a oneM2M-enabled IoT system, which monitors the CPU and memory status of each IoT node.

#### 5.2.1. Stream Query Processor Benchmark

The main goal of the benchmark test is to measure the maximum throughput and processing time of our QE with a synthetic load for a given number of simultaneous queries. We used a road traffic dataset from in Aarhus, Denmark with a range of two days (from 13 February 2014 to 14 February 2014) provided by the CityPulse project [35]. Since it has huge amount of data, we choose it as test data set for verifying the implementation of OMQ. The benchmark was conducted by injecting 100,127 vehicle count data records from 449 different roads.

We performed each experiment with three types of queries, depicted in Table 2. Each query uses 10 random data sources from 10 different roads. The benchmark was performed on two nodes: (1) a PC equipped with an intel Core i5-4460 3.20 GHz processor and 12GB RAM as an infrastructure node, and (2) a Raspberry Pi 3 single board computer equipped with an ARM-based 1.0 GHz processor as an edge/IoT node.

**Figure 13:**
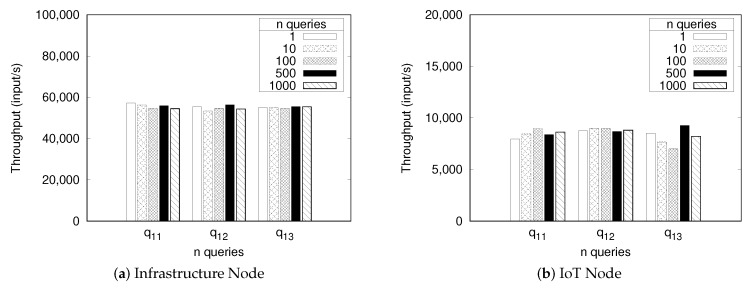
Processing throughput of stream query processor.

Figure 13 displays the benchmark results for the throughput in the infrastructure node and IoT node. The throughput in the infrastructure node reaches around 50,000–60,000 input/second in the infrastructure node and around 8000–9000 input/second in the IoT Node. The throughput stays around this range regardless of the query number and query type. The throughput of the infrastructure node always tends to be higher than that of the IoT node, since the infrastructure node has more powerful hardware.

**Figure 14:**
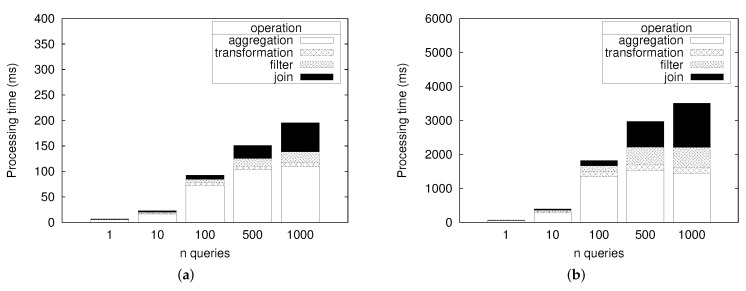
Total processing time for query $q_{13}$ for processing 100K of data. (**a**) Infrastructure Node; (**b**) IoT Node.

Figure 14 shows the total processing time needed to process all the 100,127 data records with different number of query $q_{13}$. We observe that the processing time increases with the number of queries. Most of the processing time is spent to perform aggregation, followed by join operation, filter operation, and transformation. Since the join operation can only be executed in the infrastructure node, it requires data transfer between the IoT node and the infrastructure node. Thus, the processing time for the join operation is slowest. The aggregation takes much more time than other processors except join because it could compute the final answer after seeing all input data consecutively.

The total processing time barely reaches 200 ms and 4000 ms in the infrastructure node and IoT node, respectively. However, if we expect that the throughput is calculated as the total number of records divided by the total processing time, the throughput in the first graph is very low compared to what we should expect. One possible reason for the very low throughput is the bottleneck in the input receiver module. Another reason can be the high cost needed to move data from one processor to another.

#### 5.2.2. IoT Nodes System Monitoring

**Figure 15:**
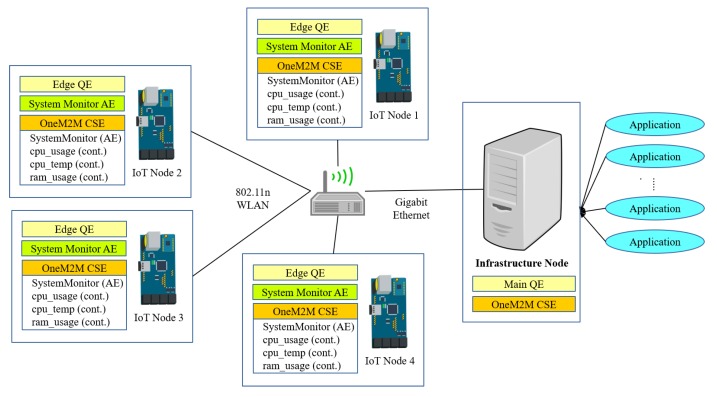
Hardware environment used for the IoT node system monitor.

The second experiment scenario consists of a oneM2M enabled IoT architecture as shown in Figure 15. Each IoT node consists of a Raspberry Pi 3 SBC (BCM2837 4 × 1.0 GHz processor, 1 GB RAM, 16 GB storage, Raspbian 20170410 OS, Linux Kernel 4.9.24-v7+) installed with our edge query engine (QE) and nCube Rosemary 2.1.14 CSE. Each IoT node also has an AE called “SystemMonitor”, which monitors the CPU usage, CPU temperature and memory usage of each node. The infrastructure node consists of an x86 PC (Intel Core i5-4460 4 × 3.20 GHz processor, 12 GB RAM, 1 TB storage, Ubuntu 16.04.3 LTS, Kernel 4.4.0-79 generic) installed with Mobius YT 2.3.8 CSE and running our main query engine (QE).

To capture an unpredictable latency, which usually happens in an IoT node, we used a Network Emulation (NETEM) program [36]. It is a tool that allows to add delay, packet loss, duplication and other characteristics to packets outgoing from a selected network interface. Thus, we can simulate a network latency in a range from 1 ms to 100 ms with a normal distribution.

In this scenario, several applications make use of the data from the IoT system. Each application issues a single stream query into the system. Table 3 lists all the queries used during the experiment. One identical query will be submitted by five different applications. We start all applications one-by-one, every 10 s, starting from the first five applications that submit the same query $q_{21}$ (denoted as $q_{21}\left(1\right)$ to $q_{21}\left(5\right)$), and continue with another five applications that submit $q_{22}$ and so on. During the application execution, several metrics are measured from each IoT node: network transfer rate, CPU usage, and memory usage. After running all the applications, the average query latency time is computed for another 10 s. All the measurements are recorded using a separate telemetry program installed on each edge device.

To see the effectiveness of our proposed system, we measure the query processing time of OM2M without QE as a baseline. This baseline time is compared to the query processing time of OM2M with QE. We use the same application programs in both cases (with and without QE). These applications execute the list of queries described in Table 3 and implemented with Node.js technology.

**Figure 16:**
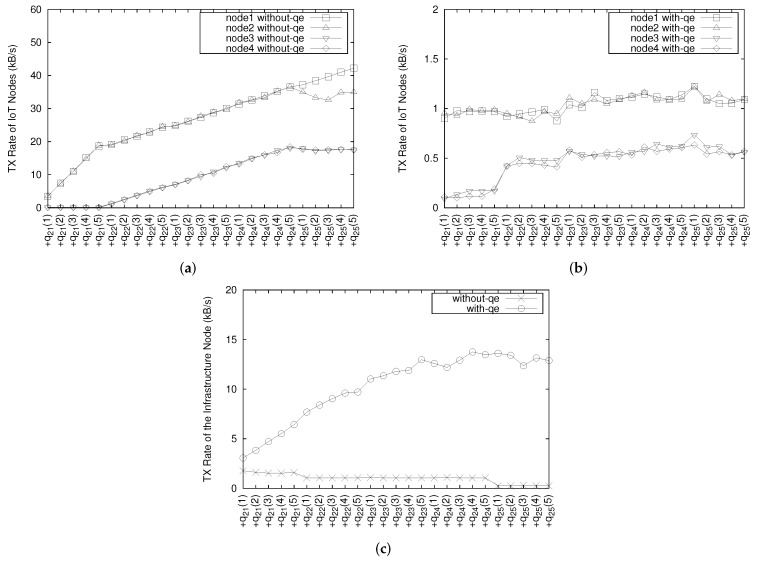
Network transfer rate. (**a**) IoT Node without QE; (**b**) IoT node with QE; (**c**) the infrastructure node.

Figure 16a–c display the network transfer rate for each IoT node and an infrastructure node. This transfer rate can be computed when OMQ sends data from each IoT node and for the infrastructure node and vice versa while it executes users queries consecutively.

When OMQ does not utilize QE (Figure 16a), we observe a gradual increase of the network transfer rates for node 1 and node 2 after running query $q_{21}$. This is because that the queries used in the experiment require transferring data from all IoT nodes. However, there is almost no utilization of the network in the infrastructure side as shown in Figure 16c. This means that all IoT nodes have to serve all applications by themselves even though the subscription is already established by all applications through the infrastructure node.

When the OMQ QE is utilized, the network transfer rates for node 1 and 2 are around 1 kB/s and the other two nodes utilize less bandwidth as depicted in Figure 16b. From Figure 16b,c, we observe that transfer rates of all IoT nodes and an infrastructure node tend to be nearly stable in the end even with increasing number of queries. This is because the QE implements a data-sharing mechanism that efficiently reuses data from the IoT nodes and shares them across many queries.

**Figure 17:**
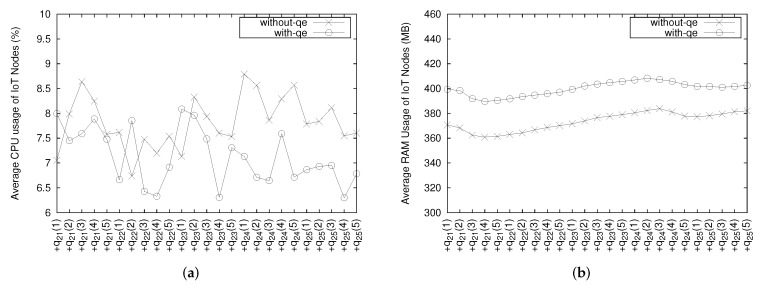
Average CPU and memory usage of all IoT nodes. (**a**) CPU usage; (**b**) Memory usage.

The average CPU and memory usage of all IoT nodes, with increase in the number of queries, are depicted in Figure 17. As we can see in Figure 17a, the CPU usage of the IoT node without QE is generally a bit higher than that of IoT node with QE. It happens because when an IoT node does not have QE to process the query operation itself, it sends the all data to an infrastructure node for further processing in the infrastructure QE. Consequently, the data communication overhead between an IoT node and an infrastructure node affects the higher CPU usage in the IoT node. However, the memory usage when implementing QE on each node is higher by about 30–40 MB as shown in Figure 17b.

**Figure 18:**
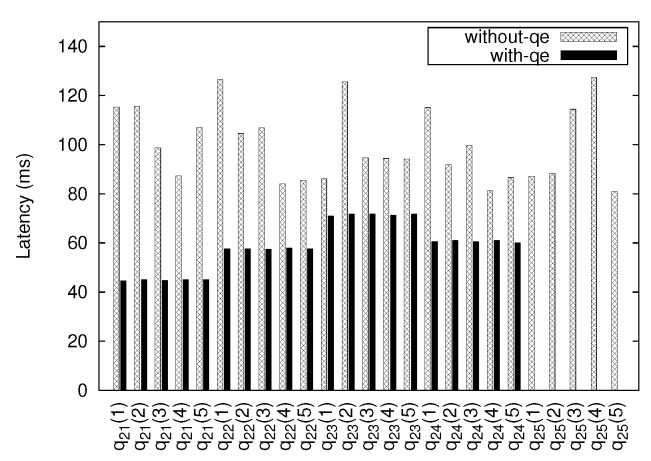
Average latency for each query type.

Figure 18 indicates the average latency of each query after running all applications simultaneously. The latency for a given query is calculated as the sum of data transfer latency and data processing time. At this case, we observe that the latency for $q_{25}$ using QE is not measured. Since the CPU temperature of IoT node 3 never exceeds the minimum threshold which specified by query $q_{25}$, the QE does not send any results to the application. Another observation is that the latency when using QE is generally lower than that without using one. One possible reason for such phenomena is that the higher the bandwidth it takes to transfer data from the IoT node, the more prone it becomes to higher network latency. The latency for each query when an application performs query without QE varies even within the same query, whereas the latency is almost the same within the same query when an application performs a query with QE because of the data-sharing mechanism.

## 6. Conclusions

In this paper, we have presented the design of OMQ for efficient retrieval of IoT data streams. The query language of OMQ enables users to retrieve IoT data from sources by specifying aggregate, filter, transform, and join operators. The OMQ supports hybrid infrastructure-edge query processing by placing the QE in two places, the first is in the infrastructure node and the second is in each IoT node. Therefore, the user queries can be processed efficiently in each IoT node rather than only in the infrastructure node, which reduces the query processing time and the network bandwidth in the infrastructure node. For efficiently processing user queries over IoT data stream, OMQ exploits the oneM2M architecture. The experimental results on real and synthetic IoT datasets indicates that OMQ can efficiently and effectively process user queries by reducing the query execution time and the usage of data bandwidth among IoT nodes. We plan to extend our OMQ bases on the following two directions: one is to enhance the query language to deal with more complex operators. The other is to design the edge deep learning algorithm for quickly obtaining analytic results from IoT data streams.

## Figures and Tables

**Table 1:** Query operators supported in OMQ.

Operator	Parameters	Description	Illustration
Aggregate ($o_{aggr}$)	Function Window Size Output Rate	Aggregate several data samples (within a range of window size) with an aggregate function such as mean, count, min, or max. Output rate determines how often the aggregation samples will be outputted regardless of the input data rate.	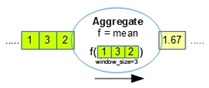
Transform ($o_{tran}$)	Function Input field Function parameters	Transform data from a given input into another form using a transformation function	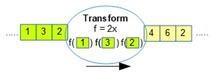
Filter ($o_{filter}$)	Filter Criteria	Filter a data input based on the given criteria	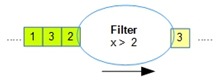
Join ($o_{join}$)	Join column Window size	Join several data stream inputs into one combined tuple. By default, the join will be performed to combine all the latest data from several inputs.	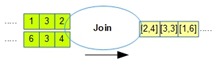

**Table 2:** List of queries for the benchmark experiment.

Query	Data Sources	Operators
$q_{11}$	Vehicle Count from 10 random places	Pre-Join 1: Aggregate − Function: Sum − Window Size: 1 h Join by timestamp
$q_{12}$	Vehicle Count from 10 random places	Pre-Join 1: Aggregate − Function: Sum − Window Size: 1 h Pre-Join 2: Transform − Function: Percentage − Max Value: 100 Join by timestamp
$q_{13}$	Vehicle Count from 10 random places	Pre-Join 1: Aggregate − Function: Sum − Window Size: 1 h Pre-Join 2: Transform − Function: Percentage − Max Value: 100 Pre-Join 3: Filter − x > 0 Join by timestamp

**Table 3:** List of queries for IoT node system monitor experiment.

Query	Goal	Data Sources	Operators
$q_{21}$	Get status of node 1 and node 2	CPU temperature of node 1 CPU temperature of node 2 CPU usage of node 1 CPU usage of node 2 RAM usage of node 1 RAM usage of node 2	Join by timestamp
$q_{22}$	Get total memory usage of all nodes	RAM usage of node 1 RAM usage of node 2 RAM usage of node 3 RAM usage of node 4	Join by timestamp Post-Join: Transform − Function: Sum
$q_{23}$	Get mean of CPU usage for every node during 2 s	CPU usage of node 1 CPU usage of node 2 CPU usage of node 3 CPU usage of node 4	Pre-Join: Aggregate − Function: Mean − Window Size: 60 s − Output Rate: 2 s Join by timestamp
$q_{24}$	Get mean CPU usage of all node every 5 s	CPU usage of node 1 CPU usage of node 2 CPU usage of node 3 CPU usage of node 4	Pre-Join: Aggregate − Function: Mean − Window Size: 60 s − Output Rate: 5 s Join by timestamp Post-join: Transform − Function: Mean All
$q_{25}$	Notify if node 3 CPU temperature is beyond 60 degree	CPU temperature of node 3	Pre-Join: Filter − x > 60
